# Cumulative Effects of Hypertension, Dyslipidemia, and Chronic Kidney Disease on Carotid Atherosclerosis in Chinese Patients with Type 2 Diabetes Mellitus

**DOI:** 10.1155/2014/179686

**Published:** 2014-04-22

**Authors:** Chuang Yuan, Christopher W. K. Lai, Lawrence W. C. Chan, Meyrick Chow, Helen K. W. Law, Michael Ying

**Affiliations:** ^1^Department of Health Technology and Informatics, The Hong Kong Polytechnic University, Hung Hom, Kowloon, Hong Kong; ^2^School of Nursing, The Hong Kong Polytechnic University, Hung Hom, Kowloon, Hong Kong

## Abstract

*Aims*. The aim of this study is to determine the extent of carotid atherosclerosis in Chinese patients with type 2 diabetes in relation to the cumulative atherosclerosis risk factors using ultrasonography. *Methods.* The presence of hypertension, dyslipidemia, and chronic kidney disease (CKD) was documented in 106 Chinese subjects with type 2 diabetes. Subjects with 0, 1, and ≥2 additional atherosclerosis risk factors were assigned into groups 1, 2, and 3, respectively (*n* = 17, 49, and 40, resp.). Using ultrasound, the carotid arteries were assessed for the presence of carotid plaque, plaque score, intima-media thickness (IMT), and carotid arterial stiffness. *Results.* With the adjustment for age and gender, the presence of plaque and plaque score were significantly higher in groups with more atherosclerosis risk factors (*P* < 0.05). In addition, age > 60 years old (odds ratio = 2.75; 95% CI: 1.26–6.0) and the presence of hypertension (odds ratio = 2.48; 95% CI: 1.11–5.58), dyslipidemia (odds ratio = 2.41; 95% CI: 1.05–5.51), and CKD (odds ratio = 7.80; 95% CI: 1.46–41.72) could independently predict higher plaque score (*P* < 0.05). *Conclusions.* Hypertension, dyslipidemia, and CKD in Chinese patients with type 2 diabetes have cumulative effects on the burden of carotid plaque.

## 1. Introduction

Carotid atherosclerosis, a chronic disease with increased carotid arterial wall thickness as well as stiffness and/or the development of carotid plaque, is associated with the occurrence of cerebrovascular events, including transient ischemia attack (TIA) and stroke. According to the World Health Organization (WHO), about 15 million of people worldwide suffer from stroke annually, and one-third of them may die and another one-third may be permanently disabled.

Type 2 diabetes mellitus (DM) is one of the major risk factors associated with carotid atherosclerosis [1]. It has been suggested that patients with type 2 diabetes have thicker and stiffer carotid arteries and are more likely to suffer from cerebrovascular events [2, 3]. In addition, atherosclerotic process in the carotid artery also associates with different traditional atherosclerosis risk factors such as hypertension [4], dyslipidemia [4], and chronic kidney disease (CKD) [5]. Moreover, these proatherosclerotic diseases are common complications in patients with type 2 diabetes [6–8]. However, it is still unclear whether the clustering of these risk factors cumulatively increases the carotid atherosclerosis burden in patients with type 2 diabetes.

Plaque, IMT, and arterial stiffness of the carotid artery are common characteristics for assessing carotid atherosclerosis. Carotid plaque narrows the lumen of the carotid artery or may rupture leading to the formation of thrombus, reducing or blocking the blood supply to the brain. Carotid plaque score has been used to quantify the severity of carotid atherosclerosis [9]. In addition, carotid intima-media thickness (IMT) and carotid arterial stiffness are structural and functional parameters of the carotid artery, respectively, which have been suggested as indicators of early carotid atherosclerosis and predictors of future cerebrovascular and cardiovascular events [10–12]. These atherosclerotic characteristics could be evaluated by ultrasonography. B-mode ultrasound is a noninvasive imaging method to assess carotid plaque and recent radiofrequency-based ultrasound technologies offer quantitative assessment of carotid IMT and carotid arterial stiffness [13, 14].

Thus, the present study aimed to investigate the cumulative effects of hypertension, dyslipidemia, and CKD on the characteristics of carotid atherosclerosis, including the presence of plaque, carotid plaque score, carotid IMT, and carotid arterial stiffness in Chinese patients with type 2 DM.

## 2. Materials and Methods

### 2.1. Subjects

In the study, subjects with type 2 diabetes were recruited from a local Chinese nonprofit making organisation for patients with diabetes (Angel of Diabetics Organisation, Hong Kong). The organisation has over 3000 registered patients who have been clinically diagnosed with DM and have regular follow-ups in diabetes clinics. Posters were put up in the premises of the Angel of Diabetics Organisation for recruitment of the patients. In the present study, the inclusion criteria of the subjects were Chinese patients with type 2 DM and older than 18 years, while the exclusion criteria of subjects were previous radiotherapy of the neck, carotid endarterectomy, and carotid stenting. Other atherosclerosis risk factors of subjects, including smoking, hypertension, dyslipidemia, and CKD, were identified with a questionnaire and a blood test. However, there was only one smoker in the 107 recruited subjects and was subsequently excluded to avoid statistical bias. As a result, a total of 106 Chinese subjects with type 2 diabetes were included in the study. The mean age of the subjects was 58.1 ± 9.0 years (ranging from 35 to 78 years) and 63.2% of them were women (*n* = 67).

This study was approved by the Human Subject Ethics Subcommittee of the Hong Kong Polytechnic University. Written consent was obtained from the subjects before the commencement of the interview and ultrasound examination.

### 2.2. Identification of Atherosclerosis Risk Factors

For each subject, the brachial blood pressure was measured with a sphygmomanometer (Tensoval, Hartmann, Germany) at the left upper arm in sitting posture after at least 10 minutes of rest. A total of 3 mL of overnight fasting blood sample was obtained and blood tests were performed by a certified medical laboratory (Bright Growth Medical Laboratory Limited, Hong Kong). The levels of blood glucose, hemoglobin A1c (HbA1c), total cholesterol, low density lipoprotein (LDL), high density lipoprotein (HDL), triglyceride, and creatinine were determined by automated clinical chemistry analyzer using reagent cartridges recommended by the manufacturer (Dimension Xpand Plus, Siemens Healthcare, Germany). Estimated glomerular filtration rate (eGFR) was defined and calculated using the CKD-EPI (chronic kidney disease epidemiology collaboration) equation [15]. Subjects were interviewed and their medical history of coronary heart disease, stroke, smoking, and medical treatments of hypertension, dyslipidemia, and CKD was obtained. The presence of smoking, hypertension, dyslipidemia, and CKD was identified with the following criteria [16–18]: smoking: current smoker consuming 10 cigarettes per day for at least six months; hypertension: blood pressure ≥ 140/90 mmHg or under hypotensive medication; dyslipidemia: fasting total cholesterol ≥ 5.2 mmol/L, low density lipoprotein ≥ 3.4 mmol/L, high density lipoprotein ≤ 1.0 mmol/L, triglyceride ≥ 1.7 mmol/L or under medication to lower level of cholesterol; CKD: eGFR < 60 mL/min per 1.73 m^2^ or with kidney damage (i.e., kidney failure).

### 2.3. Ultrasound Examinations

All ultrasound examinations were performed in a 22°C air-conditioned examination room using the Esaote MyLab Twice ultrasound unit in conjunction with a 4–13 MHz linear transducer (Esaote, Genoa, Italy). For each subject, the systolic and diastolic pressures were inputted into the ultrasound unit for evaluating carotid arterial stiffness.

Both the left and right carotid arteries were scanned and evaluated. All ultrasound examinations were performed by the same operator. The carotid arteries were assessed for the presence of carotid plaque, carotid plaque score, carotid IMT, and carotid arterial stiffness. Plaque was defined as focal thickening >50% of the adjacent intima-media layer [19]. When a plaque was identified, the plaque score was evaluated using a carotid plaque scoring system [9]. In the scoring system, the carotid artery was divided into five segments: (1) proximal common carotid (≥2 cm proximal to carotid bifurcation), (2) distal common carotid (<2 cm proximal to carotid bifurcation), (3) carotid bulb and bifurcation, (4) internal carotid artery, and (5) external carotid artery. In each segment, transverse B-mode images of the plaque were obtained and the degree of carotid artery stenosis was identified as the percentage reduction of lumen diameter at the most stenotic site. The plaque score was graded as follows: grade 0, normal or no detectable plaque; grade 1–5, at least one plaque with stenosis degree <30%, 30–49%, 50%–69%, 70%–99%, and 100%, respectively. The carotid plaque score of each subject was expressed as the summation of the scores of all segments in both carotid arteries.

**Figure 1:**
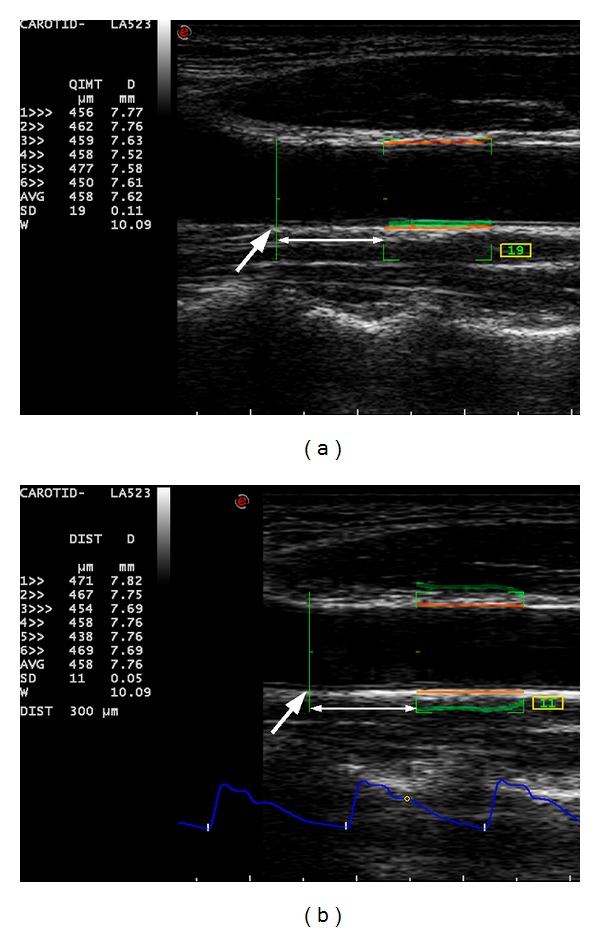
Measurements of the intima-media thickness and stiffness in the common carotid artery. (a) Longitudinal grey scale sonogram showing the measurement of the intima-media thickness of a common carotid artery (CCA) using radiofrequency-based quality intima-media thickness. (b) Longitudinal grey scale sonogram showing the measurement of the arterial stiffness of the same CCA using radiofrequency-based quality arterial stiffness. The arrows indicate the inferior end of the carotid bulb and the double-arrows lines show a distance of 1 cm.

Carotid IMT and carotid arterial stiffness were evaluated using the automated quantification programmes of the ultrasound unit: radiofrequency-based quality intima-media thickness (RF-QIMT) and radiofrequency-based quality arterial stiffness (RF-QAS), respectively (Figure 1) [14]. Carotid IMT was measured at the far wall of the common carotid artery (CCA) at a 1 cm segment 1 cm proximal to the inferior end of the carotid bulb. Carotid arterial stiffness was evaluated over the near and far walls of the CCA at the same segment (Figure 1). In the evaluation of carotid IMT and carotid arterial stiffness, the mean and standard deviation (SD) of the measurements in six consecutive cardiac cycles were automatically and continuously recorded by the system, and the mean measurement with a SD of <20 *μ*m for IMT or <30 *μ*m for stroke change in diameter for carotid arterial stiffness was obtained for data analyses. Each carotid artery was scanned three times for measuring carotid IMT and carotid arterial stiffness.

In the evaluation of carotid arterial stiffness, five arterial stiffness parameters were investigated in the study: distensibility coefficient (DC), compliance coefficient (CC), the indices of *α* and *β*, and pulse wave velocity (PWV). The lower DC and CC and the higher *α*, *β* and PWV, the stiffer the carotid artery. The equations of these parameters are

(1) $$\begin{array}{c} \text{DC}=\frac{\Delta A/A_{d}}{\Delta P}\;\left(1/\text{KPa}\right),\\ \text{CC}=\frac{\Delta A}{\Delta P}\;\left({\text{mm}}^{2}/\text{KPa}\right),\\ \alpha =\frac{A_{d}\cdot \ln\left(\text{SBP}/\text{DBP}\right)}{A_{s}-A_{d}},\\ \beta =\frac{D_{d}\cdot \ln\left(\text{SBP}/\text{DBP}\right)}{D_{s}-D_{d}},\\ \text{PWV}\;\left(\text{m}/\text{s}\right)=\sqrt{\frac{\alpha \cdot \text{DBP}}{\rho }}, \end{array}$$

where *D*_*s*_ and *D*_*d*_ mean the systolic and diastolic diameters of the artery; *A*_*s*_ and *A*_*d*_ refer to the systolic and diastolic lumen areas and SBP and DBP indicate the systolic and diastolic blood pressures; Δ*D*, Δ*A*, and Δ*P* represent the stroke change in the diameter, the lumen area of the artery, and the blood pressure, respectively; and *ρ* is the blood density.

### 2.4. Statistical Analysis

Continuous data are expressed as means ± SD. The normality of distribution was checked using Shapiro-Wilk test. The adjusted comparisons between study groups were performed using ANCOVA or logistic regression. The association between atherosclerosis risk factors of subjects and carotid plaque score were determined using ordinal regression. Paired *t*-test and Wilcoxon signed ranks test were used to compare the measurements at the left and right carotid arteries of the same individuals. All statistical analyses were performed using SPSS 20 (IBM, Armonk, New York, United States) and *P* value <0.05 was considered as significant.

## 3. Results

### 3.1. The Effects of Atherosclerosis Risk Factors on Carotid Atherosclerosis in Chinese Subjects with Type 2 Diabetes

In the 106 subjects with type 2 diabetes, the mean blood glucose and HbA1c level were 7.53 ± 1.65 mmol/L and 6.90 ± 0.96%, respectively. Among these 106 subjects, 17 subjects did not have any additional atherosclerosis risk factor (Group 1), 49 had one additional atherosclerosis risk factor (Group 2), and 40 had two or three additional atherosclerosis risk factors (Group 3). There were no significant differences of blood glucose and HbA1c level between the three groups (Table 1; *P* > 0.05).

As shown in Table 1, the age- and gender-adjusted analyses demonstrated that there were significant differences of the presence of carotid plaque between groups 1, 2, and 3 (*P* < 0.05). Subjects in groups 1, 2, and 3 had the incidence of carotid plaque of 23.5% (4 of 17), 38.8% (19 of 49), and 60.0% (24 of 40), respectively (Table 1). Subjects with more atherosclerosis risk factors had higher possibility to have carotid plaque.

Similarly, ordinal logistic regression analysis with the adjustment of age and gender showed carotid plaque score was significantly higher in groups with more atherosclerosis risk factors (Table 1; *P* < 0.05). In addition, another ordinal logistic regression model indicated that the age > 60 years old and the presence of hypertension, dyslipidemia, and CKD led to higher plaque scores in the 106 subjects (*P* < 0.05, Table 2).

In contrast, with the adjustment of age and gender of the subjects (ANCOVA), no significant differences of carotid IMT and carotid arterial stiffness were found in groups with different numbers of atherosclerosis risk factors.

### 3.2. Carotid IMT and Carotid Arterial Stiffness in the Carotid Artery with or without Plaque

In this study, it was found that the carotid artery with plaque did not have increased carotid IMT and carotid arterial stiffness. In the 106 subjects, 47 had carotid plaque while the other 59 subjects did not. After the adjustment of age and gender of subjects (ANCOVA), there were no significant differences of carotid IMT and carotid arterial stiffness in subjects with or without plaque (Table 3; *P* > 0.05). Furthermore, in the subjects with unilateral carotid plaque (*n* = 29), the IMT and arterial stiffness of the carotid artery with plaque were not significantly different from those of the contralateral carotid artery without plaque (*P* > 0.05).

### 3.3. The Left Carotid Artery* versus* the Right Carotid Artery

The results of the study suggested that the left carotid artery in subjects with type 2 diabetes was more vulnerable to atherosclerosis when compared with the right carotid artery. In the 106 subjects, there were greater carotid IMT and smaller DC (stiffer) in the left carotid artery than in the right carotid artery (Table 3; *P* < 0.05). Similarly, plaque was more commonly found in the left carotid artery for subjects with unilateral carotid plaque (total: *n* = 29 and left: *n* = 18 or 62%* versus* right: *n* = 11 or 38%).

## 4. Discussion

DM, mainly type 2 DM, is an increasing health problem worldwide. It is estimated that global diabetes adults will achieve 552 million in 2030 [20]. In China, an epidemic study found that there were 92.4 million diabetes adults and 148.2 million prediabetes adults in 2010 [21], indicating that diabetes is a major public health problem in this country to date.

DM is a common cause of atherosclerosis [1], and patients with diabetes tend to suffer hypertension, dyslipidemia, and CKD, which are also classical risk factors of atherosclerosis [6–8]. The present study found that Chinese type 2 diabetics with more additional atherosclerosis risk factors had higher incidence of carotid plaque and higher carotid plaque score. In addition, in Chinese subjects with type 2 diabetes, gender did not have significant effects on carotid plaque score, whereas the age > 60 years old and the presence of hypertension, dyslipidemia, and CKD independently predicted higher carotid plaque score (Table 2; *P* < 0.05). Among these effective predictors, CKD showed the highest relative importance in the prediction of higher carotid plaque score (odds ratio = 7.80; 95% CI: 1.46–41.72), followed by age >60 years old (odds ratio = 2.75; 95% CI: 1.26–6.0), hypertension (odds ratio = 2.48; 95% CI: 1.11–5.58), and dyslipidemia (odds ratio = 2.41; 95% CI: 1.05–5.51). It was suggested that age > 60 years old and the presence of hypertension, dyslipidemia, and CKD additively increased the presence and the score of carotid plaque in Chinese patients with type 2 diabetes. Therefore, prompt diagnoses and appropriate treatments for these atherosclerosis risk factors are necessary in Chinese patients with type 2 diabetes particularly for elder patients, and the patients with more additional atherosclerosis risk factors need more concern for atherosclerosis of the carotid artery.

However, results showed that carotid IMT and carotid arterial stiffness did not significantly increase in the groups with more additional atherosclerosis risk factors (Table 1). This result is consistent with a previous study in which diabetes and hypertension did not have additive effect on carotid thickening and stiffening [2]. The result may be due to the fact that the CCA was used for the measurement of carotid IMT and carotid arterial stiffness. The IMT and stiffness of the CCA are commonly used to represent carotid IMT and carotid arterial stiffness, but they just reflect the conditions of local carotid arterial wall or the systemic arterial wall to some extent [2]. Compared with the carotid bifurcation and bulb, the CCA is a less susceptible site to atherosclerosis [22]. Hypertension, dyslipidemia, and CKD induce abnormal shearing pressure to the endothelium, greater circulating cholesterol level, and more severe oxidative stress in blood, respectively [1]. The CCA may be less influenced by these proatherosclerotic conditions, while the bifurcation and bulb may be more susceptible to these proatherosclerotic conditions and be easier to have atherosclerotic changes. Consequently, carotid plaque, which is predominantly found in the carotid bifurcation and bulb, may not imply thickening and stiffening of the CCA, and thus measurements in the CCA may not fully reflect atherosclerotic burdens in the carotid artery. This conclusion was also supported by other findings of the present study that there were no significant differences of carotid IMT and carotid arterial stiffness between subjects with and without carotid plaque; and in subjects with unilateral carotid plaque, the carotid artery with plaque was not significantly thicker and stiffer than the contralateral carotid artery without plaque. Thus, composite evaluations, including the presence of carotid plaque, carotid plaque score, carotid IMT, and carotid arterial stiffness, may be necessary in clinical researches and practices.

The present study found that there were different atherosclerosis burdens between the left and right carotid artery in Chinese subjects with type 2 diabetes; the left carotid artery was more susceptible to carotid atherosclerosis. The result was consistent with Luo's study in which the left carotid arterial wall was thicker than the right in the elderly as well as in the subjects with atherosclerotic diseases [23]. The asymmetry between the left and right carotid arteries may be due to the anatomy of the circulation system. Normally, the left carotid artery branches from the arch of aorta while the right carotid artery branches from the innominate artery [24]. The different origins of the left and right carotid arteries induce different haemodynamics to the two arteries. If there are proatherosclerotic conditions, the asymmetric haemodynamics of the left and right carotid arteries may induce the development of carotid atherosclerosis at different degrees [23, 25]. Thus, the left carotid artery may need more concern, and carotid IMT and carotid arterial stiffness of the left carotid artery may be used to represent the atherosclerotic conditions of individuals with diabetes in future clinical researches or practices.

A limitation of this study is the small size of subjects, and thus the additive effects of atherosclerosis risk factors on carotid atherosclerosis in Chinese patients with type 2 diabetes may be not fully evaluated. Future investigations with larger sample size remain to be conducted. Because of limited number of subjects with smoking, this study did not assess the additive effect of smoking on carotid atherosclerosis in Chinese type 2 diabetics. Actually, smoking is a well-established risk factor of atherosclerosis and was shown to accentuate atherosclerosis in type 2 diabetics [26]. The only smoker in the present study also had 22% and 34% stenosis in the left and right carotid arteries, respectively. Future studies remain to be performed to comprehensively investigate the additive effect of smoking to carotid atherosclerosis in type 2 diabetics.

## 5. Conclusion

This study provides an understanding of the effects of additional atherosclerosis risk factors to carotid atherosclerosis in Chinese patients with type 2 DM. In these patients, the presence of hypertension, dyslipidemia, and CKD had cumulative effects on the burden of carotid plaque. As a result, prompt diagnoses and treatments of hypertension, dyslipidemia, and CKD are necessary for patients with diabetes, and more concern of carotid atherosclerosis should be given to the patients with more additional atherosclerosis risk factors.

## Figures and Tables

**Table 1:** Demographic and ultrasonographic characteristics in Chinese subjects with type 2 diabetes with different numbers of atherosclerosis risk factors.

Parameters	Total	Groups with different number of atherosclerosis risk factor			*P* value (Adjusted)
		Group 1 0 (*n* = 17)	Group 2 1 (*n* = 49)	Group 3 ≥2 (*n* = 40)	
Age, years	58.1 ± 9.0	56.9 ± 7.8	57.1 ± 8.9	60.0 ± 9.4	—
Gender (female/male), *n *	67/39	11/6	27/22	29/11	—
Presence of plaque, *n *	47	4	19	24	0.017*
Plaque score	—	—	—	—	0.005*
Hypertension, *n *	60	—	27	33	—
Dyslipidemia, *n *	65	—	28	37	—
CKD, *n *	5	—	0	5	—
Coronary heart disease, *n *	1	0	0	1	—
Stroke, *n *	3	0	1	2	—
Blood glucose, mmol/L	7.53 ± 1.65	7.36 ± 1.41	7.63 ± 1.85	7.47 ± 1.51	0.921
HbA1c, %	6.90 ± 0.96	6.74 ± 0.82	6.98 ± 1.05	6.85 ± 0.91	0.981
Total cholesterol, mmol/L	4.72 ± 0.85	4.38 ± 0.53	4.73 ± 0.80	4.84 ± 0.99	0.080
HDL, mmol/L	1.33 ± 0.30	1.32 ± 0.12	1.34 ± 0.34	1.32 ± 0.31	0.814
LDL, mmol/L	2.82 ± 0.73	2.54 ± 0.48	2.86 ± 0.67	2.89 ± 0.86	0.124
Triglyceride, mmol/L	1.23 ± 0.72	1.12 ± 0.48	1.16 ± 0.68	1.36 ± 0.84	0.203
eGFR, mL/min per 1.73 m^2^	87.81 ± 14.37	94.33 ± 12.67	88.66 ± 13.05	84.02 ± 15.70	0.022*
IMT, *μ*m	685.1 ± 123.3	676.7 ± 119.5	671.5 ± 128.5	705.5 ± 118.6	0.624
DC, 1/KPa	0.017 ± 0.007	0.018 ± 0.005	0.017 ± 0.008	0.015 ± 0.005	0.441
CC, mm^2^/KPa	0.781 ± 0.381	0.908 ± 0.719	0.762 ± 0.311	0.750 ± 0.280	0.343
*α*	5.590 ± 1.729	5.400 ± 1.739	5.550 ± 1.774	5.715 ± 1.703	0.866
*β*	11.302 ± 3.494	10.530 ± 3.445	11.301 ± 3.577	11.632 ± 3.446	0.633
PWV, m/s	7.986 ± 1.284	7.583 ± 1.237	7.990 ± 1.323	8.151 ± 1.250	0.434

*P value (Adjusted) indicates significant difference after the adjustment of age and gender. CKD: Chronic kidney disease; eGFR: estimated glomerular filtration rate, IMT: intima-media thickness; DC: distensibility coefficient; CC: compliance coefficient; and PWV: pulse wave velocity.

**Table 2:** The effect of atherosclerosis risk factors on carotid plaque score in subjects with type 2 diabetes (*n* = 106).

Parameters	Number of total	Plaque score			
		Odds ratio	95% CI		*P* value
			Lower	Upper	
Gender (No. of female)	67	1.22	0.55	2.71	0.634
Age (>60 years)	50	2.75	1.26	6.00	0.011*
Hypertension	60	2.48	1.11	5.58	0.027*
Dyslipidemia	65	2.41	1.05	5.51	0.037*
CKD	5	7.80	1.46	41.72	0.016*

*P value indicates significant difference. 95% CI: 95% confidence interval. CKD: chronic kidney disease.

**Table 3:** Carotid IMT and carotid arterial stiffness in subjects with type 2 diabetes.

Parameters	Subjects with type 2 diabetes					
	The subjects with plaque (*n* = 47)	The subjects without plaque (*n* = 59)	*P* value (Adjusted)	The left carotid artery (*n* = 106)	The right carotid artery (*n* = 106)	*P* value
IMT, *μ*m	706.6 ± 107.7	668.0 ± 132.8	0.171	703.3 ± 160.8	661.6 ± 129.5	<0.001*
DC, 1/KPa	0.016 ± 0.006	0.018 ± 0.007	0.758	0.016 ± 0.007	0.017 ± 0.010	0.031*
CC, mm^2^/KPa	0.768 ± 0.262	0.800 ± 0.456	0.426	0.750 ± 0.438	0.777 ± 0.353	0.111
*α*	5.930 ± 1.665	5.319 ± 1.745	0.369	5.771 ± 2.225	5.553 ± 2.425	0.132
*β*	12.066 ± 3.359	10.694 ± 3.508	0.203	11.676 ± 4.444	11.274 ± 4.898	0.153
PWV, m/s	8.162 ± 1.276	7.845 ± 1.284	0.209	8.144 ± 1.612	8.014 ± 1.721	0.265

*P value (Adjusted) indicates significant difference after the adjustment of age and gender. *P value indicates significant difference. CKD: Chronic kidney disease; IMT: intima-media thickness; DC: distensibility coefficient; CC: compliance coefficient; and PWV: pulse wave velocity.
